# PEEP titration on the basis of intratidal resistance-volume profiles

**DOI:** 10.1186/cc13463

**Published:** 2014-03-17

**Authors:** S Buehler, S Schumann, M Lichtwarck-Aschoff, J Guttmann

**Affiliations:** 1 University Medical Center Freiburg, Germany; 2 Uppsala University, Uppsala, Sweden

## Introduction

Lung-protective mechanical ventilation requires positive end-expiratory pressure (PEEP) and tidal volume (VT) to be chosen with regard to the individual state of the lung. The shape of the intratidal compliance-volume profiles might reflect the state of the lung (atelectatic, open, overdistended) and could therefore be classified into shape categories that translate into PEEP suggestions [1]. Intratidal resistance-volume profiles might reflect intratidal opening and collapse of the lung [2]. Using respiratory data from an animal study we suggest a classification into resistance shape categories based on the slope of the R(V) profiles.

## Methods

Fifteen pigs with lavage-induced lung damage were ventilated at two PEEP levels (0 and 12 mbar) and three tidal volumes (8, 12 and 16 ml/kg bodyweight). Compliance (C(V)) and resistance (R(V)) profiles for each individual animal and ventilation setting were calculated from respiratory data using the gliding-SLICE method [3]. C(V) profiles were associated with one of the six suggested shape categories. The dependency of the mean R(V) slope of all animals on the ventilation setting was used as a basis for classification into resistance shape categories. Resistance shape categories were compared with compliance shape categories for each individual animal to test whether similar PEEP suggestions result from both methods.

## Results

Small PEEP and VT were typically associated with increasing C(V), and decreasing C(V) corresponds to large PEEP and VT. A classification of each C(V) profile into one of six compliance shape categories was possible. The shapes of the R(V) profiles of individual animals were remarkably similar. The R(V) slope was typically largest for a PEEP and VT setting at which derecruitment was likely and smallest where overdistension was likely. Based on this a classification scheme was defined: 10 <slope <21 mbar s/L2 (category 1, 'increase PEEP'), slope ≥21 mbar s/L2 (category 2, 'keep PEEP') and slope ≥10 mbar s/L2 (category 3, 'decrease PEEP'). Comparison of resistance and compliance shape categories for single animals shows a good correlation.

## Conclusion

Resistance shape categories might provide additional guidance for PEEP setting. Combining compliance and resistance shape categories could improve lung-protective ventilation.
